# Functional Characterization of Friedreich Ataxia iPS-Derived Neuronal Progenitors and Their Integration in the Adult Brain

**DOI:** 10.1371/journal.pone.0101718

**Published:** 2014-07-07

**Authors:** Matthew J. Bird, Karina Needham, Ann E. Frazier, Jorien van Rooijen, Jessie Leung, Shelley Hough, Mark Denham, Matthew E. Thornton, Clare L. Parish, Bryony A. Nayagam, Martin Pera, David R. Thorburn, Lachlan H. Thompson, Mirella Dottori

**Affiliations:** 1 Murdoch Childrens Research Institute, Royal Children's Hospital, Melbourne, Victoria, Australia; 2 Department of Paediatrics, The University of Melbourne, Melbourne, Victoria, Australia; 3 Centre for Neural Engineering, Department of Electrical and Electronic Engineering, The University of Melbourne, Melbourne, Victoria, Australia; 4 Department of Otolaryngology, The University of Melbourne, Melbourne, Victoria, Australia; 5 Department of Anatomy and Neuroscience, The University of Melbourne, Melbourne, Victoria, Australia; 6 Division of Maternal Fetal Medicine, Saban Research Institute of Children's Hospital of Los Angeles, Los Angeles, California, United States of America; 7 Florey Institute of Neuroscience and Mental Health, The University of Melbourne, Melbourne, Victoria, Australia; 8 Department of Audiology and Speech Pathology, The University of Melbourne, Melbourne, Victoria, Australia; 9 Walter and Eliza Hall Institute, Melbourne, Victoria, Australia; 10 Victorian Clinical Genetics Services, Royal Children's Hospital, Melbourne, Victoria, Australia; University of Nebraska Medical Center, United States of America

## Abstract

Friedreich ataxia (FRDA) is an autosomal recessive disease characterised by neurodegeneration and cardiomyopathy that is caused by an insufficiency of the mitochondrial protein, frataxin. Our previous studies described the generation of FRDA induced pluripotent stem cell lines (FA3 and FA4 iPS) that retained genetic characteristics of this disease. Here we extend these studies, showing that neural derivatives of FA iPS cells are able to differentiate into functional neurons, which don't show altered susceptibility to cell death, and have normal mitochondrial function. Furthermore, FA iPS-derived neural progenitors are able to differentiate into functional neurons and integrate in the nervous system when transplanted into the cerebellar regions of host adult rodent brain. These are the first studies to describe both *in vitro* and *in vivo* characterization of FA iPS-derived neurons and demonstrate their capacity to survive long term. These findings are highly significant for developing FRDA therapies using patient-derived stem cells.

## Introduction

Friedreich ataxia (FRDA) is the most common form of all inherited ataxias known to date, despite it being an autosomal recessive disorder [1]. The onset of symptoms is usually observed during childhood with progressive neurodegeneration over time, resulting in severe motor dysfunction such that they can no longer walk or perform upper limb movement. Speech, vision and hearing abilities are also affected. The neurodegeneration is often accompanied by cardiomyopathy and diabetes. FRDA is caused by the presence of a trinucleotide GAA repeat expansion in the first intron of the *FXN* gene [2]. This effect is an insufficiency of the mitochondrial protein frataxin, which appears to play an important role in iron homeostasis [1], [2]. Reduced levels of frataxin protein is associated with mitochondrial dysfunction, and leads to cell toxicity and cell death [3].

Despite ongoing efforts over many years, there is still need to establish appropriate *in vivo* and *in vitro* models of FRDA to study disease pathogenesis and develop therapies. FRDA cell lines have been established from various sources, including transgenic yeast, transgenic human cell lines, patient-derived skin fibroblast lines, lymphoblastoid cell lines and primary lymphocytes [4]–[8]. These cell lines however are not representative of the cell types normally affected in the disease and are therefore somewhat limited in their use for understanding the pathological mechanisms. Cellular defects have been reported for some cell types, such as a reduction in the activity of mitochondrial protein complexes containing iron-sulfur (Fe-S) clusters in heart and skeletal muscle, and a deficit in ATP production in skeletal muscle and lymphoblasts [9]–[12]. However, these findings are not consistent across all cells, and thus, FRDA pathology appears to be contextual to different cell types [9], [13].

To address this need for cellular models of FRDA, we generated induced-pluripotent stem (iPS) cells from FRDA patient fibroblasts, referred to as FA iPS cells [14]. The FA iPS cells and their derivatives maintain the defining FRDA molecular properties of FRDA, having GAA repeat expansions on both *FXN* alleles with corresponding low expression levels of frataxin transcripts [14]. Our previous studies also demonstrated that FA iPS cells can differentiate to neurons, glia and neural crest lineages [14]. We now extend these analyses to functionally characterize neurons derived from FA iPS cells, including their electrophysiological properties, as well as mitochondrial and metabolic activities. We also assess their capacity to survive long term *in vitro* and integrate within the adult nervous system *in vivo*. These studies are fundamental for using FA iPS cell lines to establish a neuronal cellular model of FRDA, and demonstrate the potential of an iPS cell line derived from a genetically inherited neurodegenerative disease to differentiate and function *in vivo*.

## Materials and Methods

### Ethics statement

This study conformed to the Australian National Health and Medical Research Council's published Code of Practice for the Use of Animals in Research, and experiments were approved by the Florey Neuroscience Institutes animal ethics committee. All animals were housed on a 12 h light/dark cycle with *ad libitum* access to food and water.

All experiments for iPS and human embryonic stem cell lines were performed in accordance with approvals obtained from the University of Melbourne Human Ethics Committee (#0605017, #0830010 and #0829937).

### Cell culture and differentiation

IPS cells derived from FRDA patient fibroblasts (FA3 iPS and FA4 iPS) [14], control iPS cells (iPS1, WiCell USA), and the human embryonic stem cell line H9 (WA-09, WiCell USA) cell lines were cultured as previously described [15]. Briefly, mitomycin-C treated human foreskin fibroblasts in KSR media consisting of DMEM/nutrient mixture F-12, supplemented with 0.1 mM β-mercaptoethanol, 1% nonessential amino acids, 2 mM glutamine, 25 U/ml penicillin, 25 µg/ml streptomycin and 20% knockout serum replacement (all from Invitrogen). All cells were cultured at 37°C, 5% CO_2_. Colonies were mechanically dissected every 7 days and transferred to freshly prepared human embryonic fibroblasts. The FA3-GFP cells were generated by transfecting FA3 iPS cells with the *piggyBac* transposon vector (Wellcome Trust Sanger Institute) modified to contain a GFP expression cassette, driven by the human elongation factor 1 alpha promoter. Positive GFP-expressing cells were enriched by mechanical dissection of transfected FA3 iPS cells, using a Leica MZFIII Fluorescence Stereomicroscope. For neural induction, colonies were treated with 500 ng/ml human recombinant noggin (PeproTech) and 4 ng/ml bFGF in neural basal media (NBM) [16]. After 14 days, colony pieces were mechanically harvested and cultured in suspension in NBM supplemented with 20 ng/ml bFGF and 20 ng/ml EGF to promote neurosphere formation. Neuronal differentiation was performed by plating neurospheres on poly-D-lysine/laminin substrates in NBM for at least 1 week, as previously described [16].

### Electrophysiology

Neurons cultured on individual coverslips were transferred to the recording chamber mounted on the stage of an upright microscope (AxioExaminer D1, Zeiss) fitted with a 40X water-immersion objective lens. Cells were visualised with phase contrast or Dodt optics, and a monochrome CCD camera (Spot RT SE18, Diagnostic Instruments). Electrophysiological recordings in whole cell patch-clamp were performed at room temperature (22–25°C). Micropipette electrodes were fabricated from borosilicate tubing (1.0 mm O.D., 0.58 mm I.D.) using a Sutter P-2000 puller (Sutter Instrument Company) and had a tip resistance of 2–6 MW. During recordings, cells were superfused at 1–3 ml/min with extracellular buffer (137 mM NaCl, 5 mM KCl, 10 mM HEPES, 1 mM MgCl_2_, 2 mM CaCl_2_, 10 mM glucose, pH 7.35, 300–305 mosmol/kg), and micropipettes contained internal solution (115 mM K-gluconate, 10 mM HEPES, 7 mM KCl, 0.05 mM EGTA, 2 mM Na_2_ATP, 2 mM MgATP, 0.5 mM Na_2_GTP, pH 7.3, 294 mosmol/kg). Tetrodotoxin (TTX), tetraethylammonium chloride (TEA) and 4-aminopyridine (4-AP) were diluted daily to final concentrations in the bath perfusate, and administered by superfusion via a gravity-fed system. Signals were recorded with a MultiClamp 700B amplifier (Molecular Devices), and data acquisition system (Digidata 1440A, Molecular Devices), and AxoGraph X analysis software (AxoGraph Scientific). Records were digitized at 50 kHz and filtered at 10 kHz. Series resistance (*R*_S_) was monitored in response to a 10 mV voltage step (mean 18.0±0.8 MW; cells with *R*_S_ greater than 30 MW were excluded). In current clamp, pipette capacitance neutralization was applied and bridge balance utilized to compensate errors due to *R*_S_. All recordings were made from a holding potential of −73 mV, and corrections for liquid junction potential (12.8 mV; JPCalcW, courtesy of Prof. P H Barry, Sydney) were made offline. Results are presented as mean ± SEM.

### Dipstick assays for complex I activity, complex IV activity and frataxin protein

Dipstick assay for frataxin, mitochondrial complexes I (CI) and IV (CIV) were obtained from Mitosciences, and performed as per the manufactures instructions in dissociated neurospheres (day 17–19 post neuronal induction).

### TUNEL assay

Neurosphere frozen sections for TUNEL analysis were permeabilised in 0.1% Triton X-100 for 2 min on ice, followed by TUNEL labeling according to the manufacturer's protocol (Roche, *In situ* Cell Death Detection Kit, Fluorescein). Sections were photographed using an Olympus BX51 microscope and ULH100HG reflected fluorescence system with DP70 camera.

Post-image acquisition, images were rescaled from 1376 by 1038 pixels to 5504 by 4152 pixels (ImageJ, plugin TransformJ using quintic b-splines interpolation) [17]. Illumination correction was performed (CellProfiler software) [18], and contrast enhanced for each image uniformly (ImageJ, plugin CLAHE) [19]. A pixel classifier for use with CellProfiler was generated using the open source software Ilastik version 0.5 (www.ilastik.org) [20]. A CellProfiler pipeline was designed to collect intensity data from the TUNEL channel, using the areas of nuclei identified in the DAPI channel as a mask (**Figure S2**). For each nuclei identified in the DAPI channel, nuclei area, nuclei centroid location in Cartesian coordinates, integrated intensity of TUNEL, median intensity of TUNEL, and an image of the mask was collected. Data was then transformed with an arcsine transformation to approximate a normal distribution, and an ANOVA test performed.

### ATP synthesis assay

ATP synthesis rates were measured in dissociated neurospheres (day 17–19 post neuronal induction) as previously described [21], with cells permeabilised in buffer containing 0.005% digitonin with substrate/inhibitors as described (mitochondrial complex II (CII)-dependent substrate succinate (10 mM), CII inhibitor malonate (1 mM), CI-dependent substrates glutamate (10 mM), pyruvate (10 mM) and malate (10 mM), and CI inhibitor rotenone (2.5 µM)).

### Enzyme activity assays by spectrophotometry

Citrate synthase activity of dissociated neurospheres (day 19–24 post neuronal induction) was determined relative to total protein at 30°C as previously described [22].

The activity of CI was also detected spectrophotometrically in whole cell homogenates, as previously described [22]. However, due to the very large amount of error, the measurements were deemed unreliable (data not shown). This was likely due to the small amount of biological material available for analysis, and the high rotenone-insensitive rate of NADH oxidation in these samples (61%±6.3 (SEM)).

### Mitochondrial membrane potential (ΔΨ_m_)

Dissociated adherent neurospheres (day 25–33 post neuronal induction) grown in a black 96 well plate were stained in media containing 75 nM tetramethyl rhodamine methyl ester (TMRM) (Ex 544 nm, Em 590 nm) and 2 µg/ml Hoechst-33258 (Ex 346 nm, Em 460 nm) for 40 min at 37°C, 5% CO_2_. Cells were washed in warm phosphate-buffered saline (PBS), then the mitochondrial membrane potential (ΔΨ_m_) was determined as the ratio of TMRM to Hoechst fluorescence (FLUOstar OPTIMA). Control wells were treated with 20 µM carbonylcyanide-ρ-trifluoromethoxyphenyl hydrazone (FCCP) to dissipate the ΔΨ_m_.

The ΔΨ_m_ was also determined by microscopy in dissociated adherent neurospheres (day 21 post neuronal induction) grown in 35 mm glass bottom dishes (World Precision instruments). Cells were stained in Hanks Buffered Salt Solution with magnesium and calcium (Gibco) containing 75 nM TMRM for 30 min at 37°C, 5% CO_2_. Cells were visualised at 37°C, 5% CO_2_ on a Delta Vision OMX V3 Imaging System (Applied Precision) using the TRITC filter set with oil-immersion objective (Olympus, IX71), fitted with a CoolSNAP HQ^2^ camera (Photometrics). After initial imaging, 20 µM FCCP was applied to dishes to dissipate the ΔΨ_m_, and regions of interest were visualized again. Images were de-convoluted, cropped and projected with maximum intensity (Applied Precision, SoftWoRx v5.5). Mitochondrial rich perinuclear regions were selected in greater than 80 cells per cell line and average TMRM fluorescence determined pre- and post-FCCP application.

### Western blot analysis

Dissociated neurospheres (day 17–19 post neuronal induction) were analysed by western blot analysis as previously described [23], using the human OXHOS complex antibody cocktail (Abcam, MS601).

### Animals and transplantation surgery

Dissociated neurospheres derived from the FA3 GFP iPS cells were grafted into 4 adult (250 g), female athymic (nude; CBH-rnu) rats. The animals were deeply anaesthetised using 2% isoflurane as an inhalant and placed in a stereotaxic frame (Kopf) in a flat skull position. Donor neural progenitors were injected into the right cerebellar hemisphere using a 5 µl microsyringe (SGE Analytical Science) fitted with a pulled glass capillary as previously described [24]. A total of 1 µl of the cell suspension (0.5×10^5^ cells/µl) was injected over 2 min; 10.8 mm caudal to bregma; 3.6 mm lateral to the midline; and 4.0 mm below the dural surface. The cannula was left in place a further 5 min after the contents had been delivered to prevent backflow up the needle track. The animals were killed (lethal dose of pentobarbitone; intravenous perfusion) 12 weeks later for histological analysis. Animals were transcardially perfused with saline (0.9%) followed by paraformaldehyde (4% in 0.1 M PBS). The brains were removed and post-fixed for a further 2 h in 4% paraformaldehyde and cryoprotected overnight in sucrose (25% in 0.1 M PBS) before being sectioned on a freezing microtome (Leica). Parasagittal sections were collected in 12 series at a thickness of 30 µm.

### Immunohistochemistry and imaging

Immunohistochemical procedures for transplantation studies were performed as previously described in [25]. Detection of the antibody conjugates was achieved using either a peroxidase-based reaction followed by precipitation of diaminobenzidine (DAB; Sigma) or conjugation of a fluorophore. The primary antibodies and dilutions used were as follows: mouse anti-APC (1∶500; Calbiochem); rabbit anti-GABA (1∶5000; Sigma); rabbit anti-GFAP (1∶200; DAKO); chicken anti-GFP (1∶1000; AbCam); mouse anti-NeuN (1∶200; Millipore); rabbit anti-Olig2 (1∶500; Millipore); mouse anti-PSA-NCAM (1∶100; Santa Cruz); rabbit anti-Tbr1 (1∶1000; Millipore). For fluorescent labeling, secondary antibodies raised in donkey were conjugated with Alexa-488; -549 or -633 (Molecular Probes). For DAB labeling, biotin-conjugated goat anti-rabbit was used (Jackson ImmunoResearch) followed by incubation with peroxidase-conjugated streptavidin (Vectastain ABC kit, Vector Laboratories). No significant non-specific signal was generated from the secondary antibodies, as determined by control experiments where the primary antibody was omitted from the immunohistochemical procedure.

The overview of DAB-labeled GFP images were generated as a montage of single dark-field images captured using a 10X objective on a Leica DM6000 upright light microscope with the Leica X-Y tiling software module (v3.8). Fluorescent images of the grafted cells were captured using a Zeiss upright laser-scanning confocal microscope and ZEN software.

### Statistics

Unless otherwise described, error bars represent SEM, and p values are generated from student t-tests (GraphPad, PRISM V6.0b) and reported when ≤0.05.

## Results

### 1. FA iPS cells differentiate *in vitro* to functionally active neurons

We have previously described the generation and characterization of two FRDA iPS cell lines, known as ‘FA3’ and ‘FA4’, derived from adult FRDA human skin biopsies, and demonstrated their capacity to differentiate to neurons *in vitro* [14]. Here we extend these studies to determine the functionality of FA-derived neurons using whole cell patch clamp recordings. We find then that FA-derived neurons are electrically active and display core electrophysiological characteristics typical of neurons derived from human stem cells, namely *I*_Na_ and *I*_K_ [26]–[28].

**Figure 1:**
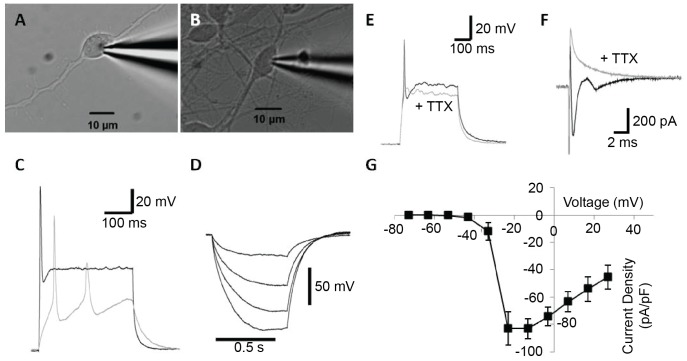
Firing properties of neurons derived from FA iPS cells. (**A**–**B**). Patch-clamp recordings were made from iPS cells displaying an ovoid soma and bipolar processes. (**C**). Action potentials were activated in response to membrane depolarization. In the example shown here, multiple action potentials are observed at threshold (gray), whilst a single spike occurs in response to supra threshold stimulation (black). (**D**). Negative current injection resulted in membrane hyperpolarisation, without the addition of a depolarizing sag or rebound action potentials at stimulus offset. (**E**). Action potentials were blocked by addition of the sodium channel antagonist TTX (gray). (**F**). In voltage-clamp recordings, the transient inward current observed during membrane depolarization (black) was abolished in the presence of TTX (gray). (**G**). I–V curve demonstrating the voltage-dependence of the TTX-sensitive sodium current.

Electrophysiological recordings were obtained for 62 FA3 iPS-derived neurons displaying an ovoid soma (∼10 mm diameter) and bipolar processes (**Figure** **1A–B**). The mean resting membrane potential was −49.5±1.3 mV, and membrane capacitance 7.7±0.3 pF. In current clamp, intracellular depolarizing current steps (20 to 190 pA; 300 ms duration) elicited action potentials in 58 of 62 (94%) of FA iPS-derived neurons (**Figure 1C**). Firing threshold was −31.8±0.7 mV, with an average action potential amplitude of 96.4±1.9 mV, and mean action potential latency of 6.8±0.3 ms and half-width of 4.8±0.4 ms during supra-threshold input (+190 pA). Of the neurons that generated action potentials, 53 displayed a single action potential fired at stimulus onset. The remaining four cells exhibited between two and four action potentials at threshold, decreasing to one or two spikes with increasing stimulation (**Figure** **1C**). Spontaneous firing in the absence of stimulation was never observed. Exposure to 1 mM tetrodotoxin (TTX), the selective blocker of voltage-gated sodium channels, inhibited firing of action potentials (**Figure 1E**). Injection of hyperpolarizing current (−5 to −30 pA) resulted in membrane hyperpolarization (**Figure** **1D**), but failed to evoke a voltage-dependent depolarizing sag indicative of a hyperpolarization-activated mixed cation current, and rebound action potentials at stimulus offset were also absent.

**Figure 2:**
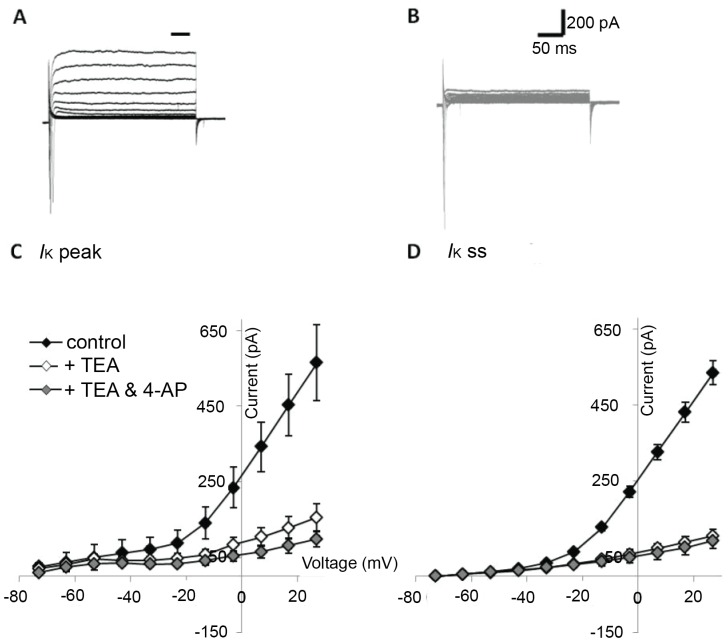
Outward currents recorded from FA iPS-derived neurons. (**A**) Membrane depolarization activates a transient sodium current followed by a sustained outward current that is attenuated by potassium channel blockers TEA (10 mM) and 4-AP (1 mM) (**B**). C–D. I–V curves depicting peak outward potassium current (**C**; *I*_K_ peak) and steady-state current (**D**; *I*_K_ ss, measured at bar in **A**) as a function of membrane potential. Currents recorded in the absence of all channel blockers (closed) were reduced in the presence of TEA (open), or TEA and 4-AP (gray).

In voltage clamp, responses measured during 10 mV increments in voltage (−63 to +27 mV; 250 ms duration) revealed a small rapidly activating and inactivating inward current (**Figure 1**) followed by a sustained outward current (**Figure 2**). The transient inward current was completely and reversibly blocked by application of 1 mM TTX (**Figure** **1F**), indicative of a sodium current (*I*_Na_). The peak TTX-sensitive *I*_Na_ was −550±73 pA (−83±7 pA/pF) at −13 mV (n = 3), measured in the presence of 10 mM TEA and 1 mM 4-aminopyridine (4-AP) (**Figure 1G**). Consistent with a potassium-mediated current (*I*_K_), the sustained outward current that followed *I*_Na_ (**Figure 2A**) was reduced by the potassium channel antagonists TEA and 4-AP (**Figure 2B**). The mean total steady-state current (*I*_K_ ss) was 535.4±31.8 pA (**Figure** **2D**), with a mean maximum peak current (*I*_K_ peak) of 565.0±33.1 pA at 27 mV (**Figure 2C**; n = 44). The steady-state portion of the slowly inactivating current was blocked by 80.2% by 10 mM TEA (n = 8), or 82.7% by 10 mM TEA and 1 mM 4-AP (**Figure 2D**; n = 5), whilst the peak current was blocked by 72.5% by 10 mM TEA (n = 8), or 82.4% by 10 mM TEA and 1 mM 4-AP (**Figure** **2C**; n = 5). Hyperpolarization of the membrane did not evoke activation of a slowly activating inward current (*I*_h_; data not shown).

### 2. Frataxin expression is reduced in FA iPS-derived neurospheres, but level of cell death is normal

**Figure 3:**
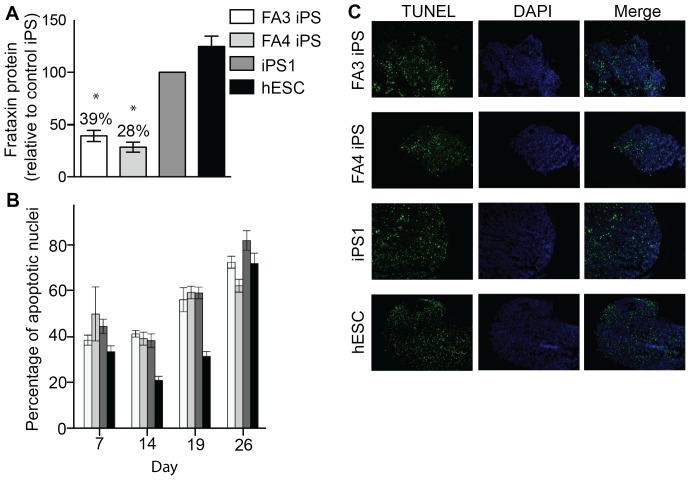
Frataxin expression and level of cell death in neurospheres derived from FA iPS cells. (**A**) Frataxin protein was measured in cell lysates using immuno-capture dipsticks and quantified relative to the control iPS line. N = 3 experiments, with at least 12 neurospheres per sample for each replicate experiment (**B**) Levels of cell death in neurospheres cultured for 7–26 days were determined by percentage of TUNEL expression. (**C**) Representative TUNEL staining images from day 26. N = 3 separate experiments per time point, with at least 5 neurospheres per time point for each experiment. *p<0.001. Error bars where shown  = SEM.

Although FA iPS cells are able to differentiate into functional neurons, the residual level of frataxin protein expression has not been quantified. Consistent though with the transcriptional expression data previously described [14], frataxin protein levels were significantly lower in both FA3 iPS-derived neurospheres (39%±5.3 SEM) and FA4 iPS-derived neurospheres (28%±4.7 SEM) relative to control iPS1-derived neurospheres (**Figure** **3A**; p<0.001). These levels correspond to the upper quartile range of frataxin protein levels found in FRDA patients, as determined in patient buccal cells [29], [30].

It is unknown however if there is cell death occurring in specific subpopulations of neurons/neural progenitors during neural differentiation that may alter findings. In order to address this, a temporal analysis of apoptotic cells was performed in neurospheres cultured for up to 26 days without passage (**Figure** **3B, C**). Following neurosphere formation both FA3 and FA4 iPS-derived neurospheres, like controls, show increasing levels of cell death from two weeks onwards (**Figure 3B**). However, at no stage is there a statistically significant increase in cell death in the FA3 and FA4 iPS-derived neurospheres compared to control iPS1 and hES-derived neurospheres (**Figure 3B**).

### 3. Mitochondrial functional analyses in FA iPS-derived neurospheres and neurons

**Figure 4:**
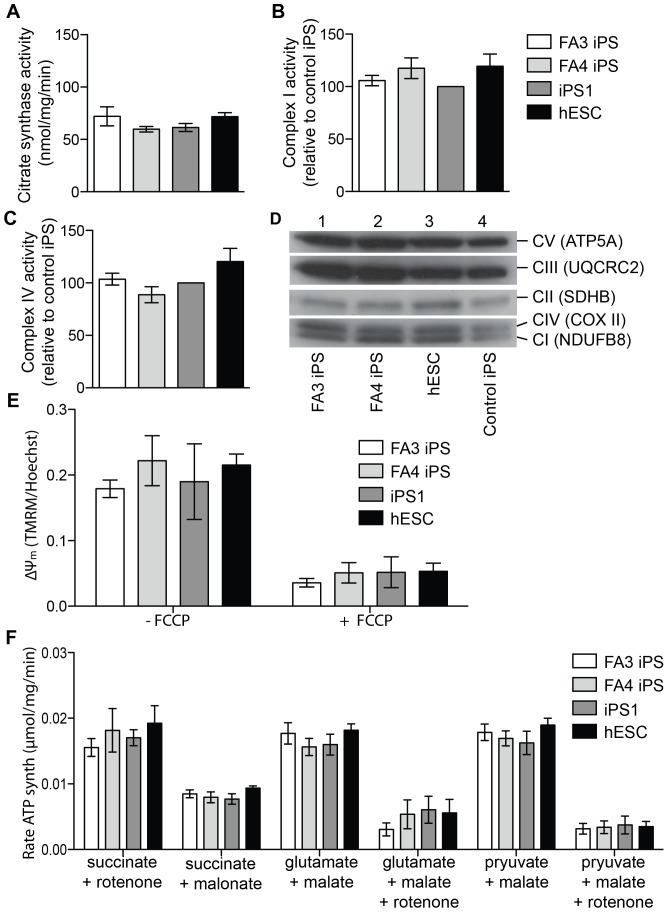
Mitochondrial functional analyses of FA iPS neural progenitors and neurons. (**A**) Citrate synthase activity was measured in neurosphere lysates spectrophotometrically and expressed relative to total protein. (**B**) CI, and (**C**) CIV activities were measured in neurosphere lysates by immuno-capture dipsticks, and expressed relative to control iPS1. (**D**) Expression of subunits representative of the OXPHOS CI-V was determined in neurosphere lysates by western blot analysis. (**E**) The ΔΨ_m_ was assessed in dissociated neurospheres plated as a monolayer by measuring the accumulation of the cationic dye TMRM relative to cell number (as determined by Hoechst staining). (**F**) The rate of ATP synthesis was measured in digitonin permeabilised neurospheres, with indicated substrate inhibitor combinations. Error bars are SEM. N = 3–5 experiments, with at least 12 neurospheres per condition for each replicate experiment.

Since FRDA is considered a mitochondrial disease, mitochondrial function was also analysed in FA iPS-derived neurospheres and from control iPS1 and hES-derived neurospheres. No significant changes were observed in citrate synthase activity in either FA3 and FA4 iPS-derived neurospheres relative to controls, indicative of no difference in mitochondrial volume per cell [31] (**Figure 4A**). The mitochondrial oxidative phosphorylation (OXPHOS) system, in which complexes I, II and III (CI, CII and CIII) contain Fe-S clusters [32], was also monitored. The activities of CI and CIV in FA3 and FA4 iPS-derived neurospheres were equivalent to control iPS1 and hES-derived neurospheres (**Figure 4B and 4C**). Expression of representative OXPHOS subunits were also unchanged in FA3 and FA4 iPS-derived neurospheres relative to controls (**Figure 4D**), suggesting no marked disruption to OXPHOS complex assembly or stability. The ΔΨ_m_ was measured by the accumulation of the cationic dye TMRM in the mitochondria [21], and was found to be unchanged in both FA3 and FA4 iPS-derived neurons (dissociated neurospheres) relative to control iPS1 and hESC-derived neurons (**Figure 4E**; **Figure S1**). Finally, CI-dependent (glutamate + malate, pyruvate + malate) and CII-dependent (succinate) ATP synthesis capacities were consistent in FA3 and FA4 iPS-derived neurospheres (**Figure 4F**) with controls. Overall, these data demonstrate that mitochondrial volume and function are not altered in FA iPS neural derivatives relative to controls.

### 4. *In vivo* differentiation potential of FA iPS-derived neural progenitors in the adult cerebellum

FA iPS derived neural progenitors and neurons do not show an overt phenotype in mitochondrial function or cell death *in vitro*. We therefore proceeded to examine *in vivo* differentiation potential of FA iPS-derived neural progenitors to determine their capacity to differentiate and integrate into the adult nervous system. These transplantation studies provide a basis to examine the *in vivo* use of FA iPS neural derivatives.

**Figure 5:**
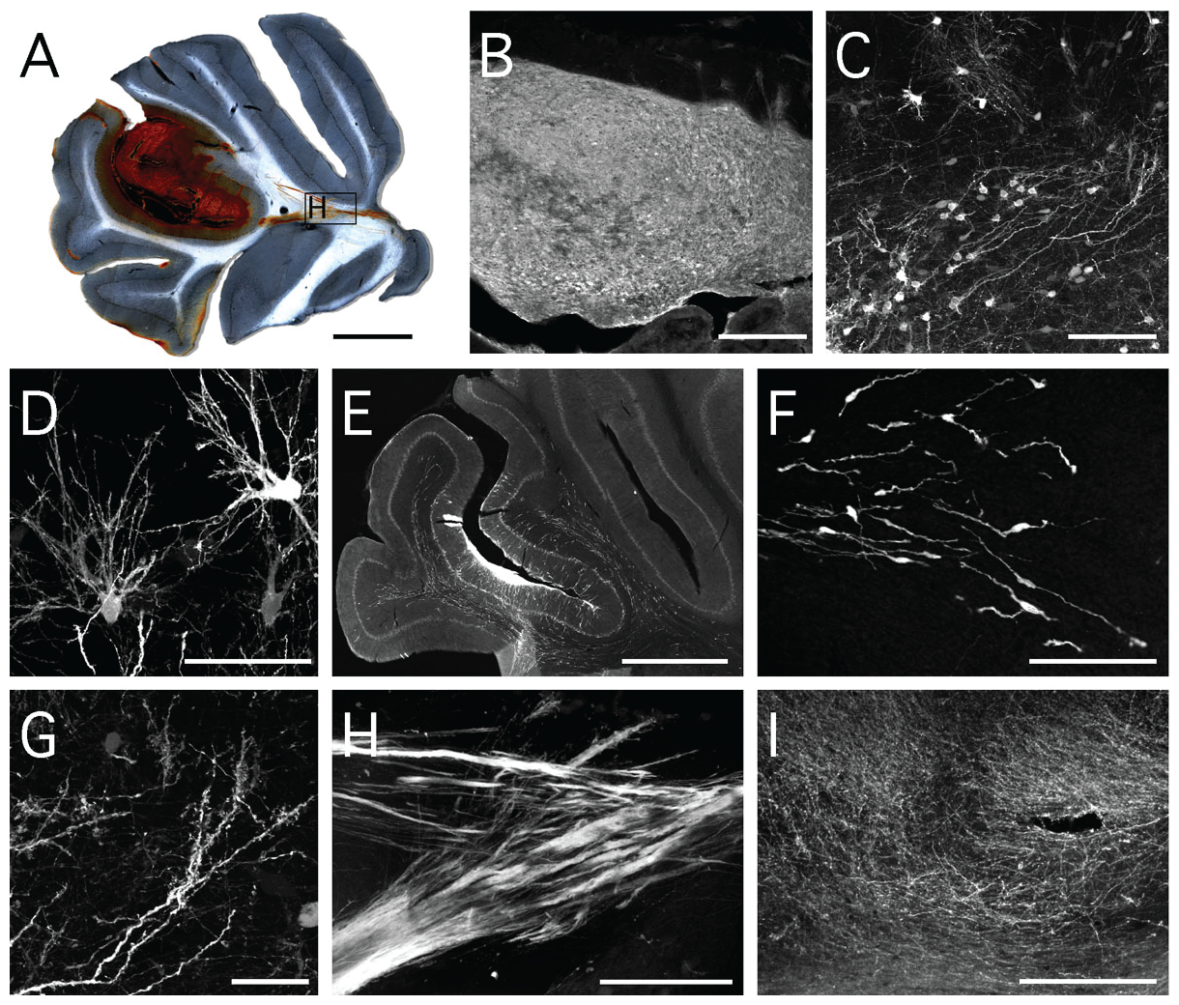
Structure and morphology of FA-iPS donor grafts revealed by immunohistochemistry for GFP. (**A**) Darkfield imaging of a representative section illustrates the size and placement of the grafts within the cerebellum. (**B**) The GFP+ cells were distributed uniformly throughout the grafts. (**C**, **D**) less densely packed cells adjacent to graft had morphological features consistent with differentiated neurons and glia. (**E**, **F**) cells with the morphology of migrating neuroblasts could be found throughout the cerebellar white matter. GFP+ fibres appeared in various forms including (**G**) spiny dendrites, (**H**) fasciculated bundles and (**I**) dense networks within the cerebellar grey matter. Scale bars: A, E - 1 mm; B, H, I – 200 µm; C, F – 100 µm; D - µm; G – 25 µm.

To detect transplanted FA cells *in vivo*, FA3 iPS cells were transduced to constitutively express GFP and then differentiated to neural progenitors. GFP-expressing FA iPS neural progenitors were transplanted into the cerebellar regions of adult rats. Immunohistochemistry for GFP at 12 weeks after transplantation revealed surviving grafts in all four animals. The grafts were positioned in the posterior lobe of the cerebellum, predominately in the grey matter, but also entering the adjacent white matter of the *arbor vitae* (**Figure** **5A**). The gross morphology of the grafts presented as discrete or contained deposits of cell mass with uniform density and no sign of overt overgrowth or tumourigenesis (**Figure** **5B**). Clusters of sparsely distributed GFP+ cells could be found in the host parenchyma, adjacent to the main graft deposit, that allowed for detailed analysis of cell morphology. Various morphological profiles consistent with both immature and terminally differentiated neural cell types, including neurons and glia, could be identified (**Figure** **5C and D**). Cells with the morphology of migrating neuroblasts (**Figure** **5F**) were distributed extensively throughout the white matter of the grafted hemisphere (**Figure** **5E**). The mitochondrial location of GFP and consequent widespread distribution throughout cells allowed also for detailed resolution of graft derived fiber patterns. Many single GFP+ fibers appeared as spiny dendrites (**Figure** **5G**), consistent with integration at the synaptic level. Large fasciculated fiber bundles were observed originating from the graft and traversing on an anterior pathway through the white matter and deep cerebellar nuclei (**Figure** **5H**), however, no GFP+ fibers were observed outside of the cerebellum. Dense networks of GFP+ fibers were also seen throughout the cerebellar grey matter (**Figure** **5I**).

**Figure 6:**
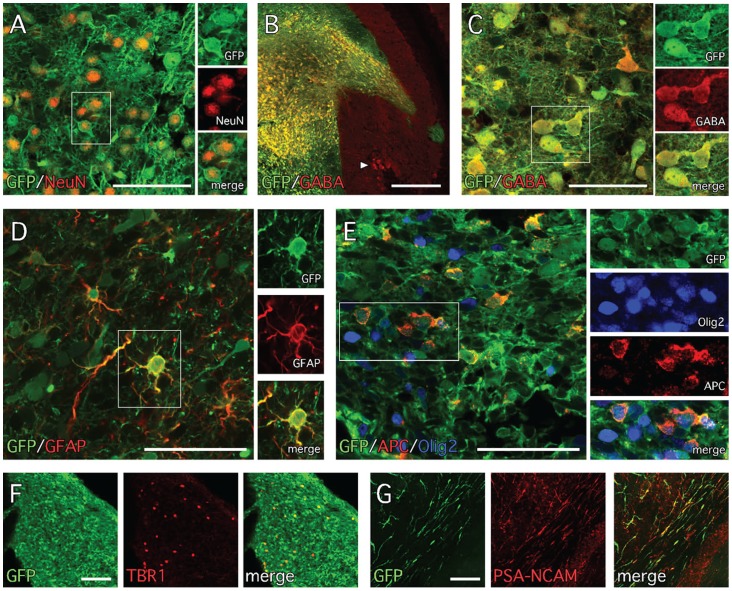
Neuronal phenotypes of grafted FA-iPS neural progenitors. (**A**) The grafts were rich in differentiated neurons based on immunohistochemistry for GFP and NeuN. (**B**, **C**) Many of the neurons also expressed the neurotransmitter GABA (arrowhead in B shows host GABA+ Purkinje neurons). GFP+ cells with glial phenotypes were also present based on immunohistochemistry for (**D**) the astrocyte marker GFAP and (**E**) the oligodendrocyte markers Olig2 and APC. (**F**) A small population of GFP+/TBR1+ cells were scattered throughout the graft core. (**G**) The majority of the GFP+ cells with the morphology of migrating neuroblasts also expressed the cell adhesion molecule PSA-NCAM. Scale bars: A, C, D, E – 50 µm; B – 200 µm; F, G – 100 µm.

Double labeling for antigenic markers of cell phenotype revealed the presence of GFP+ cells with neuronal or glial identity as well as migrating neuroblasts. The grafts appeared rich in neurons based not only on morphology but also on the presence of large numbers of cells expressing the neuronal nuclear marker NeuN (**Figure** **6A**). The vast majority of GFP+ neurons in the grafts also expressed the inhibitory neurotransmitter γ-aminobutyric acid (GABA; **Figure** **6B and C**). Cells with antigenic features of glia were more sparsely distributed in the grafts and included GFAP+ astrocytes as well as cells expressing markers consistent with immature (Olig2+/APC-) and mature (Olig2+/APC+) oligodendrocytes (**Figure** **6D and E**). Another sparsely distributed population of cells expressed the transcription factor Tbr1 (**Figure** **6F**). The cells with morphology of migrating neuroblasts, distributed widely throughout the white matter, were found to express the cell adhesion molecule PSA-NCAM (**Figure** **6G**). Taken together, FA iPS-derived neural progenitors show a robust capacity to integrate and differentiate into neuronal and glial lineages within the adult nervous system.

## Discussion

In FRDA, there is severe degeneration within the nervous system, particularly in cerebellar neurons and peripheral sensory neurons [1]. However, over time, more widespread neurodegeneration is observed within patients that affect their vision, speech, hearing and fine movement, suggesting that multiple neurological pathways may be involved in disease pathogenesis. The aim of this study was to characterize neural progenitors and neurons derived from FA iPS cells in order to confirm their capacity to differentiate into functional neurons. Furthermore, we sought to examine whether FA iPS-derived neurospheres demonstrated any altered sensitivity to cell death or changes in mitochondrial function, thereby reflecting disease pathogenesis.

Previously we showed that FA iPS were able to differentiate to neural progenitors with a similar efficiency as control iPS cells [14], and we now show that these FA iPS-derived neurons are capable of firing action potentials and demonstrated sodium and potassium current activity, consistent with other stem cell-derived neural progenitors [26]–[28]. Also, there was no difference observed in cell survival in FA iPS neurospheres compared to control hES/iPS-derived neurospheres. In keeping with these results, there was also no evidence that mitochondrial function or volume were altered in FA iPS neural derivatives. These results suggest that in standard culture conditions, FA neural progenitors and neurons do not show any substantial disadvantage in their long-term survival or mitochondrial function.

The lack of an overt phenotype in FA iPS neural derivatives is consistent with the notion that obvious symptoms of this disease are often observed in the later childhood years of patients [33]. Further, there is a correlation between GAA repeat expansion length, frataxin protein levels and disease severity in patients [11], [29]. Symptomatic FRDA patients generally have over 50 bp GAA repeats on each *FXN* allele, and 5–35% frataxin protein levels [29], [30]. Although FA3 and FA4 iPS-derived neurospheres retain over 200 bp GAA repeat expansions on each allele, their levels of frataxin protein are only reduced to 39% and 28%, respectively, relative to iPS control neurospheres. Therefore, we postulate that the frataxin levels in the FA iPS cells and their neural derivatives may not be low enough to demonstrate any significant phenotype in mitochondrial and cellular function. Another explanation may be that the FA cells need to be activated, or exposed to increased stress in their microenvironments to induce degenerative cellular responses, since it has been reported that cell lines deficient in frataxin protein are more sensitive to stress [34]. This is an area of ongoing investigation for us. Alternatively, mitochondrial function may appear normal in our FA iPS-derived neurospheres as FRDA is a progressive disease, and cellular dysfunction may only accrue over a longer time period then we were able to analyze in our model system. Finally, another possible reason for the lack of phenotype may be that degenerative mechanisms are predominantly occurring in specific neuronal subpopulations, rather than universally in all neurons, similar to what is observed in patients. Further studies then are required to determine how FA iPS-derived neurons may display disease-related cellular dysfunction, such as the propensity for iron accumulation [35], [36]. This subsequently needs to be properly assessed by showing a reversal of phenotype upon increasing frataxin levels within the cells. Such studies are needed for establishing a neuronal model system to study FA pathogenesis.

To date, four independent groups, including our laboratory, have published the generation of FRDA iPS cells [14], [37]–[39]. Of these, only Puccio and colleagues reported a reduced ΔΨ_m_ and morphological evidence of mitochondrial degeneration in FRDA iPS-derived neurons and cardiomyocytes, respectively [37]. The authors showed that frataxin mRNA expression levels in FRDA iPSCs were reduced to 30–35% of controls, however relative levels of frataxin protein in the neuronal derivatives were not reported. These studies highlight that FRDA iPS cells may demonstrate aspects of FRDA degeneration, however the severity may be variable between different iPS lines given the different genetic backgrounds, GAA-repeat expansions, frataxin protein levels, and culture conditions.

This is the first study though to show transplantation of FA iPS-derived neural progenitors into the adult nervous system. Of significance, the site of transplantation was in the cerebellum, a predominant site of degeneration in FRDA. FA iPS donor cells showed robust differentiation to both neuronal and glial lineages, and interestingly, FA iPS-derived neurons extended their fasciculated neurites along endogenous white matter tracts. These results demonstrate the capacity of FA iPS neural derivatives to survive, differentiate, and structurally integrate into the adult nervous system. Future studies will be required to analyze their potential to establish appropriate synaptic connectivity with host neurons and form functional circuits within existing networks. These studies support feasibility for the use of patient-derived stem cells for transplantation into the nervous system as a therapeutic strategy to treat FRDA, whether it is for cell replacement and/or as vehicles to secrete factors that may promote regeneration.

## Conclusions

Despite not showing an obvious phenotype, neurons derived from FA iPS cells are still highly useful for screening candidate compounds that may increase frataxin protein levels and not induce neurotoxicity. Our studies also demonstrate that neurons may not require relatively high levels of frataxin protein for their normal functionality, which is a promising result in terms of designing therapeutic strategies needed to increase frataxin levels in FRDA patients. In summary, FA iPS cells are highly valuable for establishing a human cellular model system of FRDA that can be further utilised to accelerate development of FRDA treatments.

## Supporting Information

Figure S1 **Mitochondrial membrane potential in FA iPS-derived neurons.** (**A**) The ΔΨ_m_ was assessed by microscopy in dissociated neurospheres plated as a monolayer by measuring the accumulation of the cationic dye TMRM in the mitochondrial rich peri-nuclear region. Greater then 80 cells from a single dish pre-incubation and post-incubation with the protonophore FCCP were analysed with representative images in (**B)**.
(TIF)

Figure S2 **Indicative images for TUNEL staining, and post image acquisition analysis in FA iPS-derived neurons.** Indicative images are shown for the TUNEL assay depicting: the TUNEL stain; DAPI stain; the ‘nuclei’ mask that is generated from the DAPI stained image; and the merged ‘nuclei’ and TUNEL montage.
(TIF)
