# Supplementary figures and images for: Functional Characterization of Friedreich Ataxia iPS-Derived Neuronal Progenitors and Their Integration in the Adult Brain
Source: PLoS One. 2014 Jul 7;9(7):e101718. doi: 10.1371/journal.pone.0101718 (PMC4084949; doi:10.1371/journal.pone.0101718)

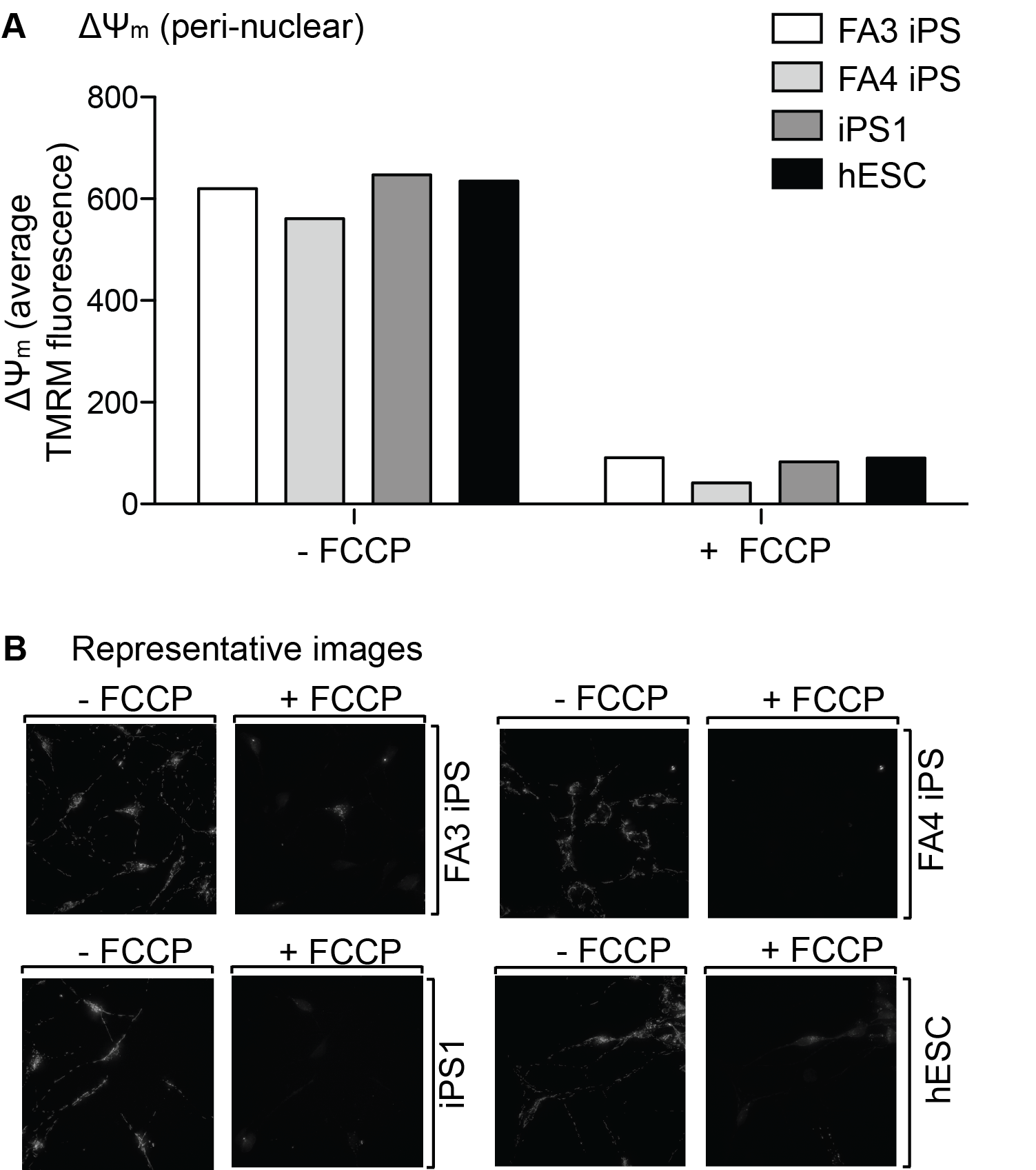

Supplement: Figure S1 — Mitochondrial membrane potential in FA iPS-derived neurons. (A) The ΔΨm was assessed by microscopy in dissociated neurospheres plated as a monolayer by measuring the accumulation of the cationic dye TMRM in the mitochondrial rich peri-nuclear region. Greater then 80 cells from a single dish pre-incubation and post-incubation with the protonophore FCCP were analysed with representative images in (B). (TIF) [file pone.0101718.s001.tif]

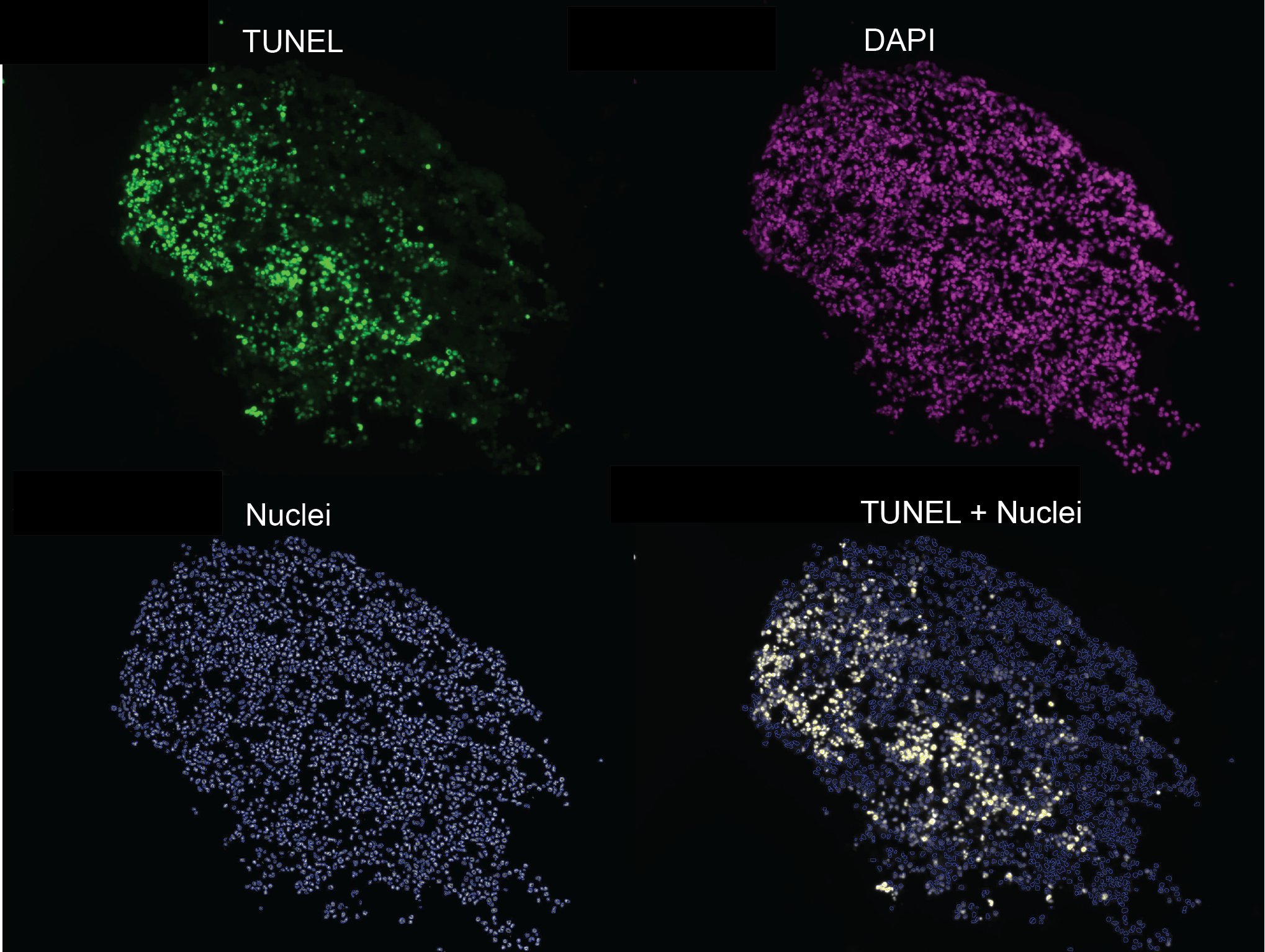

Supplement: Figure S2 — Indicative images for TUNEL staining, and post image acquisition analysis in FA iPS-derived neurons. Indicative images are shown for the TUNEL assay depicting: the TUNEL stain; DAPI stain; the ‘nuclei’ mask that is generated from the DAPI stained image; and the merged ‘nuclei’ and TUNEL montage. (TIF) [file pone.0101718.s002.tif]
